# Genomic insights into the mechanisms of FGFR1 dependency in squamous cell lung cancer

**DOI:** 10.1172/JCI174171

**Published:** 2023-11-01

**Authors:** Netta Mäkinen, Matthew Meyerson

**Affiliations:** 1 Department of Medical Oncology, Dana-Farber Cancer Institute, Boston, Massachusetts, USA.; 2 Cancer Program, Broad Institute of Harvard and MIT, Cambridge, Massachusetts, USA.; 3 Departments of Genetics and Medicine, Harvard Medical School, Boston, Massachusetts, USA.

## Abstract

Although subsets of patients with lung squamous cell carcinoma (LSCC) benefit from immunotherapy, there are few effective molecularly targeted treatments for LSCC. Fibroblast growth factor receptor (FGFR) inhibitors provide a therapeutic option for patients with LSCC harboring FGFR aberrations, but their therapeutic efficacy has been limited to date. In this issue of the *JCI*, Malchers et al. identified tail-to-tail rearrangements, either within or near *FGFR1*, that are associated with FGFR1 dependency and sensitivity to FGFR inhibition in LSCC. These results may help improve the selection of patients with LSCC who are most likely to benefit from treatment with FGFR inhibitors.

## Treatment of LSCC remains an unmet need

Lung squamous cell carcinoma (LSCC) is the second most common subtype of non–small cell lung cancer (NSCLC) after lung adenocarcinoma. In the past decades, notable advances have been made in understanding the molecular genomic landscape of NSCLC, which has paved the way for the development of effective molecularly targeted therapies, such as tyrosine-kinase inhibitors (TKIs) for activating mutations in the epidermal growth factor receptor (*EGFR*) gene and anaplastic lymphoma kinase gene (*ALK*) rearrangements (1–3). While these therapies have substantially improved the survival of patients with lung adenocarcinoma, they have been largely ineffective against LSCC because of its distinct molecular profile, leading to a widening divide in the management of these two lung cancer subtypes (4).

LSCC is a heterogeneous malignancy associated with smoking and characterized by a high mutational burden, which is already present in the early stages of the disease (5, 6). Currently, the first-line systemic treatment options for advanced LSCC include chemotherapy and immune checkpoint inhibitors, which are administered as monotherapy or combination therapy (7). Although the use of immune checkpoint inhibitors has improved the overall survival of patients with LSCC, many patients remain ineligible for this first-line treatment option. For example, only approximately 23% to 30% of patients with advanced NSCLC have sufficiently high programmed cell death ligand 1 (PD-L1) expression levels to qualify for the use of pembrolizumab (8–10). Also, mutations in HLA genes that are frequently observed in LSCC can render the patients unresponsive to immunotherapy (11).

Thus, identification of targeted therapies and reliable predictive molecular biomarkers is essential for the effective management of LSCC. The marked genomic complexity and lack of clear oncogenic drivers in LSCC have led researchers to focus their efforts on various signaling pathways that are frequently mutated in this disease to identify attractive and actionable therapeutic targets. For example, the high rate of genomic alterations in the fibroblast growth factor receptor (FGFR) signaling pathway in patients with LSCC (5, 12, 13) has made FGFR inhibitors a promising therapeutic option for this lung cancer subtype.

## Limited response to FGFR-specific small-molecule inhibitors

The FGFR family has four members, FGFR1–4, each of which consists of an extracellular region with three immunoglobulin-like domains, a single hydrophobic membrane-spanning segment, and a cytoplasmic tyrosine kinase domain. These receptors participate in the regulation of multiple biological processes, including cell proliferation, differentiation, migration, and survival (14). Abnormal FGFR signaling associated with FGFR aberrations has been observed in various cancer types, including urothelial bladder carcinoma, cholangiocarcinoma, and NSCLC (15). In recent years, the FDA has approved various FGFR-specific small-molecule inhibitors via their Accelerated Approval Program for the treatment of metastatic urothelial bladder carcinoma (erdafitinib [JNJ-42756493], objective response rate [ORR] 40%) and advanced unresectable cholangiocarcinoma (pemigatinib [INCB054828], ORR 36%; infigratinib [BGJ398], ORR 23%) based on encouraging results from clinical trials (16–18).

*FGFR1* amplification at 8p11 is the main type of FGFR alteration in LSCC, occurring in approximately 20% of patients (5). Also, various other types of genomic alterations in FGFR family members have been reported in a smaller subset of patients with LSCC, including somatic activating *FGFR2* and *FGFR3* mutations (6%) and chromosomal rearrangements leading to *FGFR3-TACC3* gene fusions (0.6%) (12, 13). Most *FGFR2* and *FGFR3* mutations in LSCC affect the extracellular region of the protein, while FGFR3-TACC3 fusion proteins have been shown to retain the FGFR3 kinase domain and its activity.

*FGFR1* amplification was originally proposed to be a predictive biomarker of FGFR inhibition in advanced LSCC based on promising results from preclinical in vitro and in vivo studies. For example, inhibition of FGFR signaling using both nonselective FGFR TKIs and FGFR-specific small-molecule inhibitors resulted in growth suppression and induced apoptosis in LSCC cell lines with *FGFR1* amplification (19, 20). Furthermore, *FGFR1*-amplified LSCC xenograft models showed impaired tumor growth when treated with FGFR inhibitors (21, 22). Consequently, *FGFR1* amplification has been a key inclusion criterion for phase I/II clinical trials in patients with advanced LSCC. It is worth noting, however, that *FGFR1* amplification did not always predict a response to FGFR inhibitors in the preclinical studies (19, 20, 22).

To date, several FGFR-specific small-molecule inhibitors have been tested in phase I/II clinical trials in patients with advanced LSCC (23). Most clinical trials, however, have indicated that *FGFR1* amplification is not a reliable predictor of response to FGFR inhibitors, with overall response rates of 8%–11% (23). The discrepancy between *FGFR1* amplification status and the clinical response to FGFR-specific small-molecule inhibitors highlights the need to better understand the mechanisms of FGFR1 dependency to identify patients most likely to benefit from these inhibitors.

## Potential mechanisms of FGFR1 dependency

**Figure 1:**
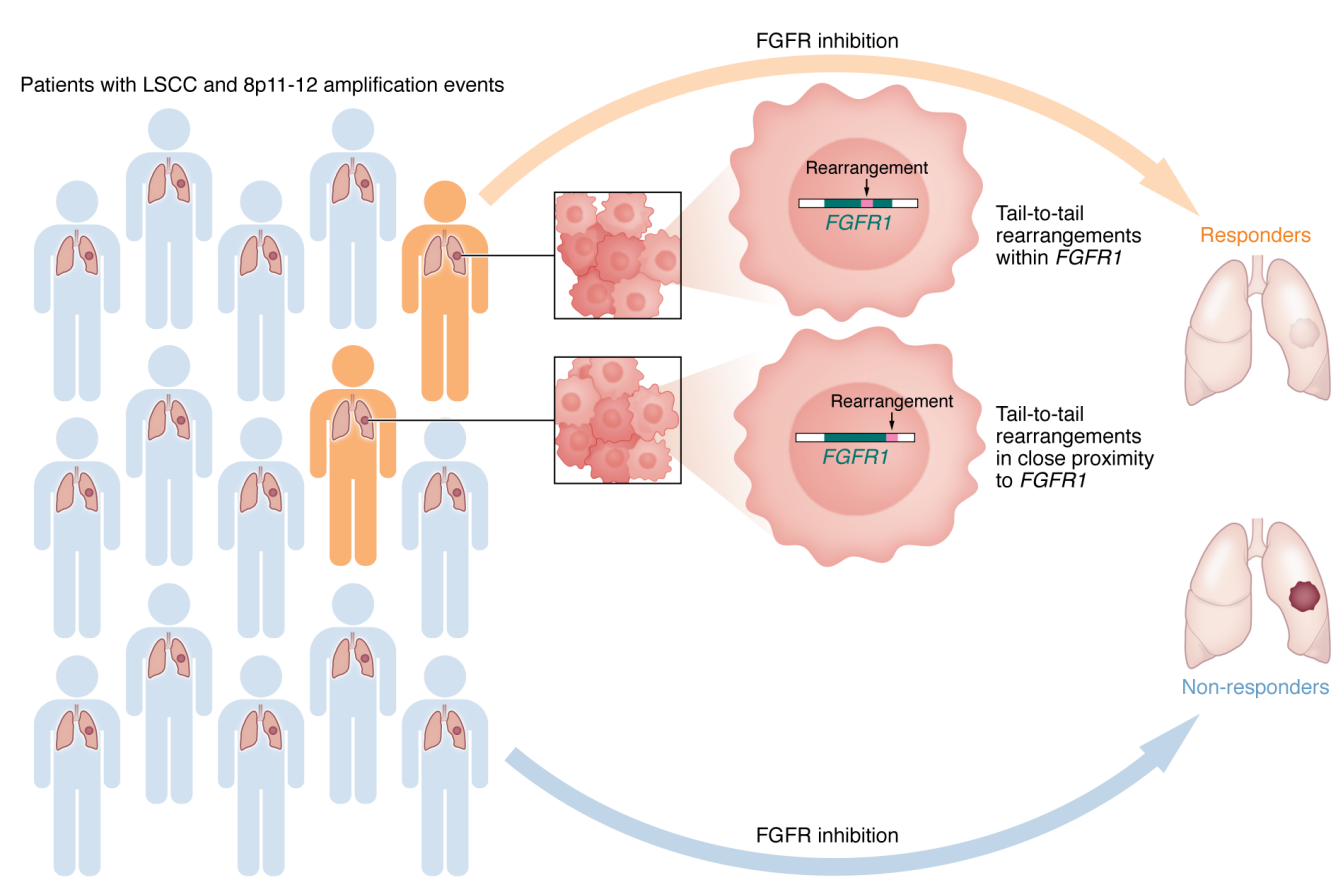
Two types of *FGFR1* rearrangements are associated with sensitivity to FGFR inhibition in LSCC. Only a subset of patients with LSCC characterized by amplification of the 8p11-12 region, which houses the putative *FGFR1* oncogene, respond to FGFR inhibition. Malchers et al. showed that LSCC tumors with intragenic tail-to-tail rearrangements within FGFR1 and in close proximity to FGFR1 were associated with FGFR1 dependency (24). Screening patients for these rearrangement events may identify those more likely to benefit from treatment with FGFR inhibitors.

In this issue of the *JCI*, Malchers and authors performed a detailed genomic characterization of *FGFR1*-amplified LSCC samples to further study the mechanisms of FGFR1 dependency (24). These efforts expand on their previous finding of marked heterogeneity among the 8p11-12 amplification events in LSCC due to the presence of multiple centers of amplification in the chromosomal region (25). *FGFR1* was observed to locate in the epicenter of the amplicon in only 28% of all 8p11-12–amplified cases. In their current article, Malchers et al. describe two types of genomic alterations that are associated with FGFR1 dependency and, thus, sensitivity to FGFR inhibition: tail-to-tail rearrangements within *FGFR1* and in close proximity to *FGFR1* (24) (Figure 1).

Tail-to-tail rearrangements within *FGFR1* were identified in 8% (4 of 52) of the *FGFR1*-amplified LSCC samples, leading to various deletions in the extracellular region of the protein. Half of the rearrangements were present in patients with a partial response to FGFR inhibition (infigratinib or pazopanib) and the other half in untreated patients. Interestingly, FGFR1 ectodomain truncations resulted in retained protein expression and catalytic function of FGFR1 in the affected tumors. Additionally, the truncated FGFR1 variants were shown to be oncogenic and sensitive to FGFR inhibition (infigratinib and AZD4547) both in vitro and in vivo. Also, tail-to-tail rearrangements in close proximity to *FGFR1* were observed in three samples unresponsive to FGFR inhibition (24).

Tail-to-tail rearrangements in close proximity to *FGFR1* were observed in 27% (14 of 52) of *FGFR1*-amplified LSCC samples. Four alterations were present in either lung cancer cell lines or patient-derived xenograft models sensitive to FGFR inhibitors (infigratinib or AZD4547), one in a patient with a partial response to FGFR inhibition, and nine in untreated patients. These rearrangements led to an *FGFR1*-centered amplification pattern in the samples and frequently co-occurred with disruptive rearrangements of *NSD3* (64%; 9 of 14), a neighboring gene of *FGFR1*. No tail-to-tail rearrangements in close proximity to *FGFR1* were observed in samples unresponsive to FGFR inhibition (24).

In summary, Malchers and colleagues identified recurrent tail-to-tail rearrangements within and in close proximity to *FGFR1* in 8p11-12–amplified LSCC samples sensitive to FGFR inhibition. They propose that these specific rearrangements could be predictive of therapeutically relevant FGFR1 dependency. Not all the study findings by Malchers and co-authors, however, fit neatly under this assumption. Two *FGFR1*-amplified LSCC samples that responded to FGFR inhibitors (22%; 2 of 9) did not harbor FGFR1 ectodomain–deficient variants or *FGFR1*-centered amplifications, although one of the samples did display a disruptive *NSD3* rearrangement (24).

Failure of *FGFR1* amplification to reliably predict a response to FGFR inhibition in LSCC has raised the possibility that genes other than *FGFR1* in the 8p11-12–amplified region could be driving or contributing to tumorigenesis. A recent study on *NSD3* reproduced previous studies nominating this gene as an oncogenic driver in LSCC and suggested that NSD3 dependency renders LSCC therapeutically vulnerable to bromodomain inhibition (26). Malchers and authors observed disruptive *NSD3* rearrangements only in FGFR inhibitor–sensitive samples (56%; 5 of 9) and proposed that sensitivity to FGFR inhibition in these samples was primarily driven by FGFR1, whereas the role of NSD3 in these tumors was unlikely functional (24). They also showed that bromodomain inhibition had no effect on the viability of Ba/F3 cells expressing ectodomain-deficient FGFR1 (24).

## Perspectives of FGFR inhibition in LSCC

To date, no FGFR-specific small-molecule inhibitors have been approved by the FDA for the treatment of LSCC patients with FGFR aberrations, given the modest therapeutic efficacy of these inhibitors in early-phase clinical trials. One of the main challenges has been the appropriate identification of patients most likely to benefit from FGFR inhibitors, which has primarily been based on *FGFR1* amplification events. Because *FGFR1* amplification status alone does not seem to robustly predict drug sensitivity, additional biomarkers have been considered, including *FGFR1* mRNA and protein expression, increased FGF ligand availability, and activation of downstream signals. Only limited clinical data for these additional biomarkers are currently available, and thus their response to FGFR inhibitors remains elusive. However, it is noteworthy that a stronger correlation between *FGFR1* mRNA and protein expression than between *FGFR1* amplification and FGFR1 expression has been described in LSCC (20, 27).

A better understanding of the pathogenesis of *FGFR1*-amplified tumors is essential to improve the selection of patients and increase the success rate of FGFR inhibitors in the clinic. The findings by Malchers et al. (24) indicate that new approaches are likely needed to test LSCC patients with *FGFR1* amplification for ectodomain-deficient FGFR1 variants and *FGFR1*-centered amplifications. Depending on the availability of the tissue sample, targeted DNA/RNA-based high-throughput sequencing platforms could be used to identify *FGFR1*-centered and focal amplification patterns.

Other indications and potential predictive biomarkers for FGFR inhibition in LSCC include somatic activating *FGFR* mutations and gene fusions. For example, mutations in the extracellular region of *FGFR2* and *FGFR3* in LSCC samples have been shown to drive cellular transformation and respond to FGFR inhibition in preclinical settings (28). Recently, a patient with LSCC who had an *FGFR3-TACC3* gene fusion was successfully treated and retreated with erdafitinib (29). *FGFR3-TACC3* gene fusions have also emerged as a potential mechanism of resistance to EGFR inhibitors (30). However, given the paucity of clinical data, which mainly stem from individual LSCC cases and a few clinical trials, the predictive value of *FGFR* mutations and gene fusions as biomarkers in LSCC needs to be further studied.

In conclusion, identification of clinically relevant predictive biomarkers for FGFR inhibition in patients with LSCC has been challenging. The detailed genomic profiling of *FGFR1*-amplified LSCC by Malchers et al. (24) provides insights into FGFR1 dependency, supporting further clinical exploration of FGFR-specific small-molecule inhibitors as a targeted therapy for FGFR1-driven LSCC.

## Version 1 11/01/2023

Electronic publication
